# Supplementary material for: A Small Molecule That In Vitro Neutralizes Infection of SARS-CoV-2 and Its Most Infectious Variants, Delta, and Omicron
Source: Biomedicines. 2023 Mar 15;11(3):916. doi: 10.3390/biomedicines11030916 (PMC10046252; doi:10.3390/biomedicines11030916)
Supplement: Supplementary file 1 [file biomedicines-11-00916-s001.zip › biomedicines-2172259-supplementary.pdf]

Supplementary information

Material and Methods

Molecular Modeling

Following well established protocols in summary; structures (PDB 6M0J, 6VXX, and 6VYB) were used to prepare models of the SARS-Cov-2 Spike protein RBD and the ACE-2 receptor. CD04872SC was initially expanded into its 3D minimized structure using BALLOON 3-1.6.6 and then processed with PRODRG4 to build topology files. Both the spike protein and the ligand were prepared for docking analysis using AUTODOCK-TOOLS and AUTODOCK_VINA 1.1.2. Docking for each of the millions of compounds evaluated from the Maybridge and ZINC library was completed with exhaustiveness set to 100 and the top 20 poses evaluated in each tranche and evaluated in both the Microsoft Azure Cloud and our university servers.

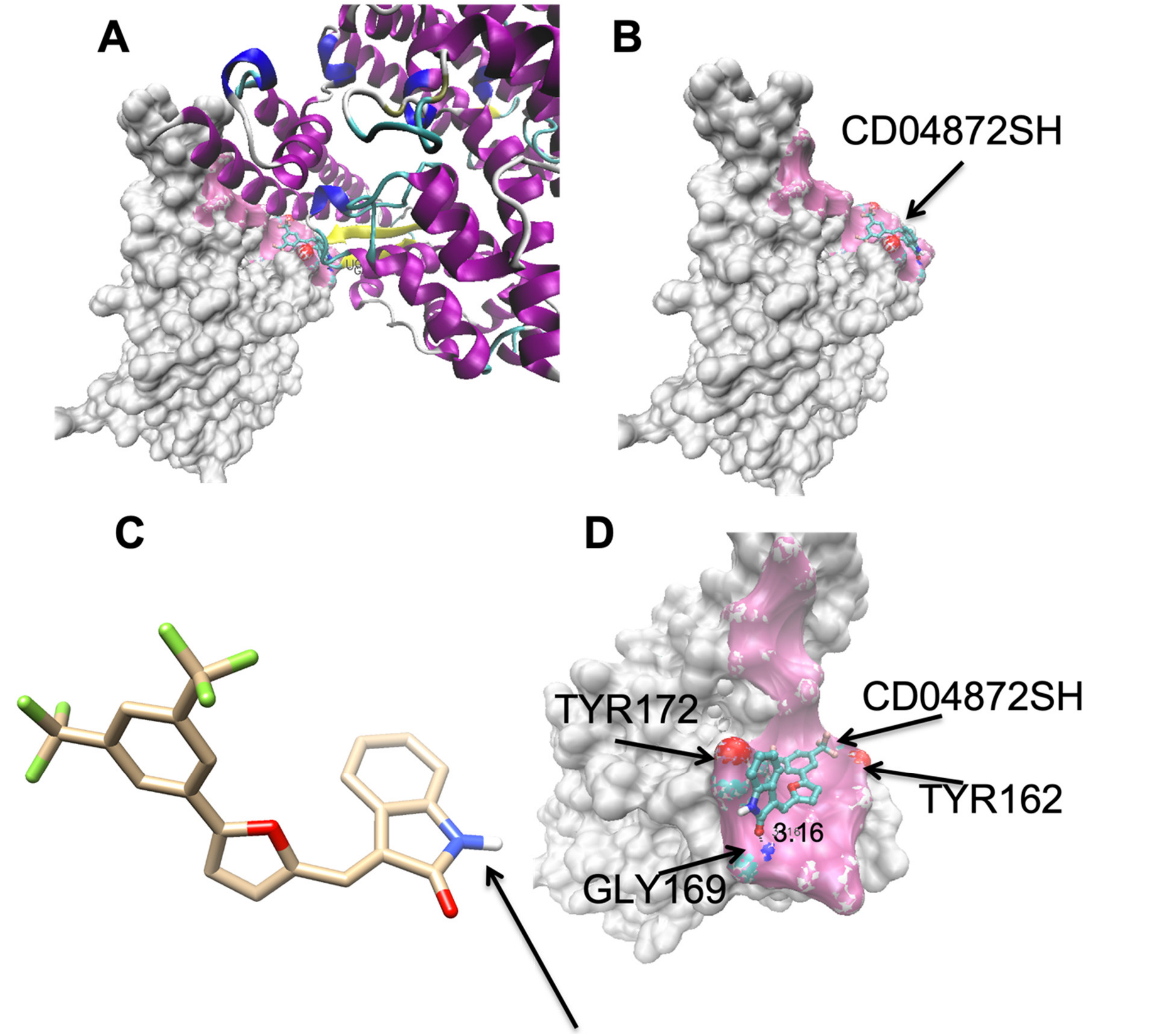

Supplementary Figure S1. 3D Schematic and mechanistic representation of our approach to disrupt the interface between the SARS-CoV2 Spike protein and the ACE2 receptor. A) Spike RBD (white surface) / Human ACE2 receptor complex. B) Pink surface is the interface with CD04872SC bound on the face. C) CD04872SC 3D structure represented in stick. D) CD04872SH has a 3.1 Armstrong Hydrogen-bond with backbone N of GLY169 and hydrophobic interactions with TYR116, TYR162, and TYR172. CD04872SC (formula $C_{24}H_{10}O_2N_1F_6$).

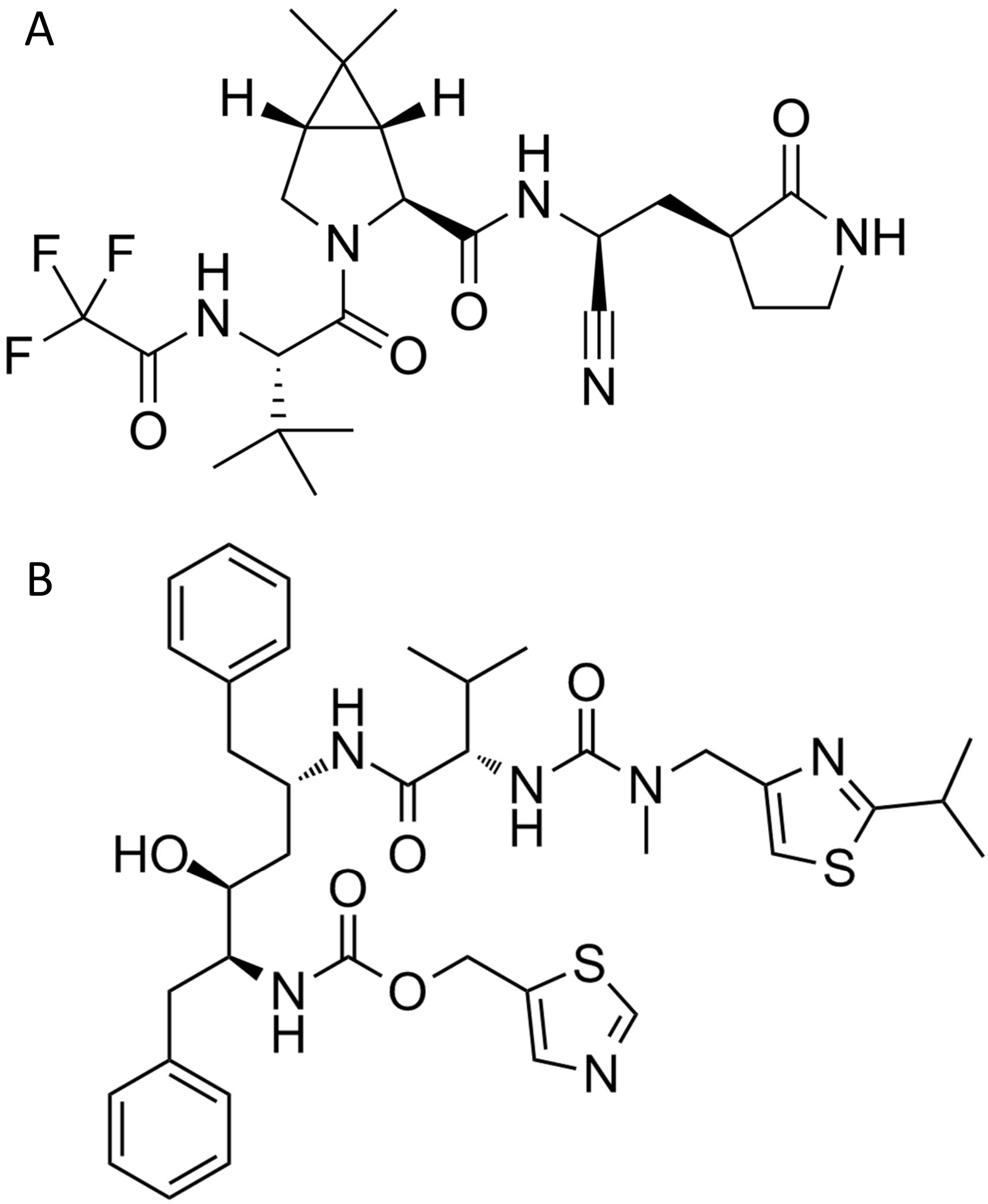

Supplementary Figure S2. 2D structure of the COVID-19 treatment recently developed by Pfizer and sold under the name of Paxlovid. It consists of two small molecules: A) Nirmatrelvir (PF-07321332) and B) Ritonavir.

Lentivirus-mediated Expression of the Spike Protein of SARS-CoV2

All manipulations were always taking place in a biosafety cabinet. HEK293 cells were transfected with the plasmids containing SARS-CoV-2, Wuhan-Hu-1 (GenBank: NC_045512), spike-pseudotyped lentiviral kit (NR-52948, from Bei Resources) designed to generate pseudotyped lentiviral particles expressing the spike (S) glycoprotein gene, as well as luciferase (Luc2) and ZsGreen. Seventy-two hours after transfection, the medium was collected in a 50 ml tube and store at -80 ^{o}C for further applications. This protocol only requires Biosafety Level 1 (BSL1) conditions and the viruses used in this protocol were replication defective.

The Plasmid expressing the human ACE2 gene was aquired from Addgene (Cat. No. 1786). Plasmid expressing the Delta Spike Protein (Cat. No. VG40819-UT) from Sino Biological.

And the plasmid expressing the Omicron Spike Protein (Cat. No. MC_0101274) was from GenScript.

In the following lines, we show a detailed protocol for Generation of Pseudotyped Lentiviral Particles by transfecting HEK293. We used the following protocol from Crawford and Coworkers[1]:

We seeded HEK293 cells in DMEM High Glucose growth medium so that they will be 70% confluent the next day. For a six-well plate, this is 8×10^5 cells per well. The next morning 12-16 h after seeding, transfect the cells with the plasmids required for lentiviral production. We transfected using Viafect (Promega Corporation, Cat. No. E4982) following the manufacturer's instructions and using the following plasmid mix per well of a six-well plate (plasmid amounts should be adjusted for larger plates):

- 1µg of lentiviral backbone–ZsGreen (NR-52520) or the Luciferase-IRES-ZsGreen (NR-52516) backbone.

- 0.22 µg each of plasmids HDM-Hgpm2 (NR-52517), pRC-CMV-Rev1b (NR-52519), and HDM-tat1b (NR-52518).
- 0.34µgviralentry protein—either SARS-CoV-2 Spike (NR-52513, NR-52514)
- At 60h post transfection, collect virus by harvesting the supernatant from each well and filtering it through a 0.45 µm SFCA low protein-binding filter. Then viruses were concentrated by using Lenti-X ™ (Cat. No. 631231, Takara Bio Inc). Virus was resuspended in 200µl of PBS. Viral suspensions were stored frozen at −80 ◦C.

Neutralization Assay

We seeded a poly-L-lysine-coated 96-well plate with 4 × 10^4 HEK293 cells per well in 100 µL DMEM High Glucose. The following morning, we transfected the cells with Viafect (Promega, Cat. No. E4982) and 100ng of ACE2 plasmid per well.

We let the ACE2 receptor express in the cells for 36h after transfection. Then, we pretreated the cells with different concentrations of the different compounds and incubated the cells for 1h.
Then we used 10µl concentrated viral suspension to infect the cells in the 96 well plate. We incubated at 37 ◦C for 36h before reading out green fluorescence (530/30 filter to detect ZsGreen in the FITC channel). For our analysis, we first subtracted out the background signal (average of the "virus only" and "virus + HEK293" wells) and then calculated the "maximum infectivity" for each plate as the average signal from the wells without drug ("virus + cells" wells). Then we calculated the "fraction infectivity" for each well, as the green fluorescence reading from each well divided by the "maximum infectivity" for that plate. For the curves shown in Figure 1, we then fit and plotted the fraction infectivity data using the Prism 7. This program fits a three-parameter Hill curve to determine the potency of neutralization (equation 1), with the top baseline being a free parameter and bottom baseline fixed to zero.

Supplementary Table S1.

Molecule	CD04872SC
Canonical SMILES	O=C1Nc2c(/C/1=C\c1ccc(o1)c1cc(cc(c1)C(F)(F)F)C(F)(F)F)cccc2
Formula	C21H11F6NO2
MW	423.31
#Heavy atoms	30
#Aromatic heavy atoms	17
Fraction Csp3	0.1
#Rotatable bonds	4
#H-bond acceptors	8
Theorical redox potential	0.7
#H-bond donors	1
MR	99.84
TPSA	42.24
iLOGP	3.27
XLOGP3	5.43
WLOGP	8.1
MLOGP	4.32
Silicos-IT Log P	6.48
Consensus Log P	5.52
ESOL Log S	-6.04
ESOL Solubility (mg/ml)	3.85E-04
ESOL Solubility (mol/l)	9.10E-07
Ali Log S	-6.07
Ali Solubility (mg/ml)	3.58E-04
Ali Solubility (mol/l)	8.45E-07
Silicos-IT LogSw	-8.84
Silicos-IT Solubility (mg/ml)	6.12E-07
Silicos-IT Solubility (mol/l)	1.45E-09
GI absorption	Low
BBB permeant	No
Pgp substrate	Yes
CYP1A2 inhibitor	Yes
CYP2C19 inhibitor	Yes
CYP2C9 inhibitor	No
CYP2D6 inhibitor	No
CYP3A4 inhibitor	No
log Kp (cm/s)	-5.03
Lipinski #violations	1
Ghose #violations	1
Veber #violations	0
Egan #violations	1
Muegge #violations	1
Bioavailability Score	0.55
PAINS #alerts	0
Brenk #alerts	1
Leadlikeness #violations	2
Synthetic Accessibility	3.39

*All these theorical parameters were calculated according to reference 6.

Statistics and Reproducibility

The results were analyzed using the Prism 7 application (Graph Pad Software Inc., San Diego, CA). Dose–response curves were fitted using the following three-parameter equation:

$$Response = Bottom + \frac{Top - Bottom}{1 + 10^{(logEC_{50} - \log[A])}} \qquad (1)$$

Where Bottom and Top are the lower and upper plateaus, respectively, of the concentration–response curve, [A] is the molar concentration of the agonist, and EC50 is

the molar concentration of agonist required to generate a response halfway between the top and the bottom.

References:

1. K. H. D. Crawford, R. E., A. S. Dingens, A. N. Loes, K. D. Malone, C. R. Wolf, H. Y. Chu, M. A. Tortorici, Veesler, M. Murphy, D. Pettie, N. P. King, A. B. Balazs and J. D. Bloom. Protocol and Reagents for Pseudotyping Lentiviral Particles with SARS-CoV-2 Spike Protein for Neutralization Assays. *Viruses* **12**, 1-15 (2020).
2. Structure of the SARS-CoV-2 spike receptor- binding domain bound to the ACE2 receptor. Jun Lan, Jiwan Ge, Jinfang Yu, Sisi Shan, Huan Zhou, Shilong Fan, Qi Zhang, Xuanling Shi, Qisheng Wang, Linqi Zhang & Xinquan Wang. Nature https://doi.org/10.1038/s41586-020-2180-5.
3. Structure, Function, and Antigenicity of the SARS- CoV-2 Spike Glycoprotein. Alexandra C. Walls, Young-Jun Park, M. Alejandra Tortorici, Abigail Wall, Andrew T. McGuire, David Veesler. 2020, Cell 180, 1–12.
4. D Bakthavatsalam, J.W. Craft Jr., A Kazansky, N Nguyen, G Bae, A.R. Caivano1, C.W. Gundlach IV, A Aslam, S Ali, S Gupta, S.Y. Lin, H.D. Parthiban, P Vanderslice, C.C. Stephan and D.G. Woodside; Identification of Inhibitors of Integrin Cytoplasmic Domain Interactions With Syk; Front. Immunol., 08 January (2021) 10.3389/fimmu.2020.575085
5. P Cherubin, MC Garcia, D Curtis, CB Britt, J.W. Craft Jr., H Burress, C Berndt, S Reddy, J Guyette, T Zheng, Q Huo, B Quiñones, JM Briggs, K Teter. Inhibition of Cholera Toxin and Other AB Toxins by Polyphenolic Compounds. PLoS One. Nov 9;11(11), (2016)
6. A. Daina and V. Zoete. A BOILED-Egg to predict gastrointestinal absorption and brain penetration of small molecules. *ChemMedChem* (2016) 11(11):1117-1121.
